# On the Complexity of Human Neuroanatomy at the Millimeter Morphome Scale: Developing Codes and Characterizing Entropy Indexed to Spatial Scale

**DOI:** 10.3389/fnins.2017.00577

**Published:** 2017-10-18

**Authors:** Daniel J. Tward, Michael I. Miller

**Affiliations:** Center for Imaging Science, Department of Biomedical Engineering, Kavli Neuroscience Discovery Institute, Johns Hopkins University, Baltimore, MD, United States

**Keywords:** computational anatomy, diffeomorphometry, neuroimaging, anatomical prior, entropy, complexity, rate distortion

## Abstract

In this work we devise a strategy for discrete coding of anatomical form as described by a Bayesian prior model, quantifying the entropy of this representation as a function of code rate (number of bits), and its relationship geometric accuracy at clinically relevant scales. We study the shape of subcortical gray matter structures in the human brain through diffeomorphic transformations that relate them to a template, using data from the Alzheimer's Disease Neuroimaging Initiative to train a multivariate Gaussian prior model. We find that the at 1 mm accuracy all subcortical structures can be described with less than 35 bits, and at 1.5 mm error all structures can be described with less than 12 bits. This work represents a first step towards quantifying the amount of information ordering a neuroimaging study can provide about disease status.

## 1. Introduction

The trend toward a quantitative, task based, understanding of medical images leads to the simple goal of answering “how many bits of information would one expect a medical image to contain about disease status?” Knowing the answer to this question could impact a clinician's decision of whether or not to order an imaging study, particularly in the case where it involves ionizing radiation. This quantity can be studied in terms of mutual information between disease status and anatomical form.

(1) $$\begin{array}{r} MI\left(\text{disease},\text{ anatomy}\right)=H\left(\text{anatomy}\right)-H\left(\text{anatomy}|\text{disease}\right) \end{array}$$

where *MI* is mutual information, and *H*(·) is entropy and *H*(·|·) is conditional entropy.

In general, the higher the complexity of a population of normal anatomy, the less informative is a realization as manifest by an MRI concerning some disease. On the other hand, the simpler the class of anatomy, the more information gained by making an MRI. This is reflected by sensitivity and specificity of statistical tests.

Other information theoretic quantities could have a direct impact on clinical decision making as well. The inverse of the Fisher information puts a lower bound on the variance of any unbiased estimator (the Cramér-Rau inequality). The Kullback-Leibler divergence *D*(*P*_1_||*P*_2_) between two probability distributions *P*_1_ and *P*_2_ can be used to quantify bounds on error rates (false positives or false negatives) for any statistical test (Sanov's theorem). More specifically, for a fixed false positive rate, the false negative rate is bounded by exp(−*nD*(*P*_1_||*P*_2_)) for sample size *n*. In the typical setting of “multivariate normal, common covariance Σ, different means μ_1_, μ_2_,” this quantity is given by $D\left(P_{1}||P_{2}\right)=\frac{1}{2}{\left(\mu_{1}-\mu_{2}\right)}^{T}\Sigma^{-1}\left(\mu_{1}-\mu_{2}\right)$, a well known signal to noise ratio related to linear discriminant analysis.

To begin applying the powerful machinery of information theory to the study of anatomical form, we turn our attention to the quantity at the heart of information theory: the entropy. We propose a new method for quantifying the entropy of human anatomy at clinically relevant spatial resolutions, biological organization at the millimeter or *morphome* scale (Hunter and Borg, 2003; Crampin et al., 2004). In this work we focus our attention on developing this method and quantifying entropy for a single population, leaving inferences about specific populations or disease states to future work.

Since Shannon's original characterization of the entropy of natural language in the early 50's, the characterization of the combinatoric complexity of natural patterns such as human shape and form remains open. Human anatomical form, unlike word strings in English, are essentially continuum objects, extending all the way to the mesoscales of variation. Therefore, computing the entropy subject to a resolution, or measurement quantile becomes the natural approach to quantifying the complexity of human anatomy. Rate-distortion therefore plays a natural role. The distortion measure is played by the resolution, and in this paper we introduce the natural resolution metric that any anatomist or pathologist would use in examining tissue which would be the sup-norm distance in defining the boundary of an anatomical structure.

This paper focuses on these issues, calculating what we believe is the first bound on the complexity of human anatomy at the 1 mm scale. 1 mm seems appropriate since so much data is available via high throughput magnetic resonance imaging (MRI) and therefore that scale of data becomes ubiquitously available. Also so many studies of neuroanatomy and psychiatric disorders today are focused on the anatomical phenotype at this scale.

While the entropy of human anatomy seems difficult to define, the theory of Kolmogorov complexity gives us a precise tool for describing arbitrary objects in such a manner. The complexity of any object, which is related to its entropy by an additive constant, can be defined as the length of the shortest computer program that produces it as an output. As discussed in Cover and Thomas (2012), this quantity generally cannot be computed; doing so would be equivalent to solving the halting problem. However, any example of such a program serves as an upper bound on complexity. In what follows we describe our approach, which will serve as one such upper bound.

Our approach is to follow on Kolmogoroff's beautiful theory for calculating complexity of subcortical neuroanatomy by demonstrating codebooks that attain given logarithmic sizes coupled to a computer program which decodes elements of the codebook and attain the distortion measure. We also calculate various rate-distortion curves showing the trade off in complexity as a function of distortion.

The field of computational anatomy (Miller et al., 2014) has been developing the random orbit model of human anatomy, where a given realization can be generated from a template (a typical example of an anatomical form) acted on by an element of the diffeomorphism group. Such diffeomorphic transformations can be generated from an initial momentum *vector* (i.e., closed under linear combinations) though geodesic shooting (Miller et al., 2006). Our work has largely focused on brain imaging and neurodegenerative diseases, and we therefore carry out an examination of subcortical gray matter structures. By using a sparse representation of initial momenta supported on anatomical boundaries, and learning Bayesian prior models for initial momenta from large populations (Tward et al., 2016), we can produce an efficient representation of anatomical form.

**Figure 1:**
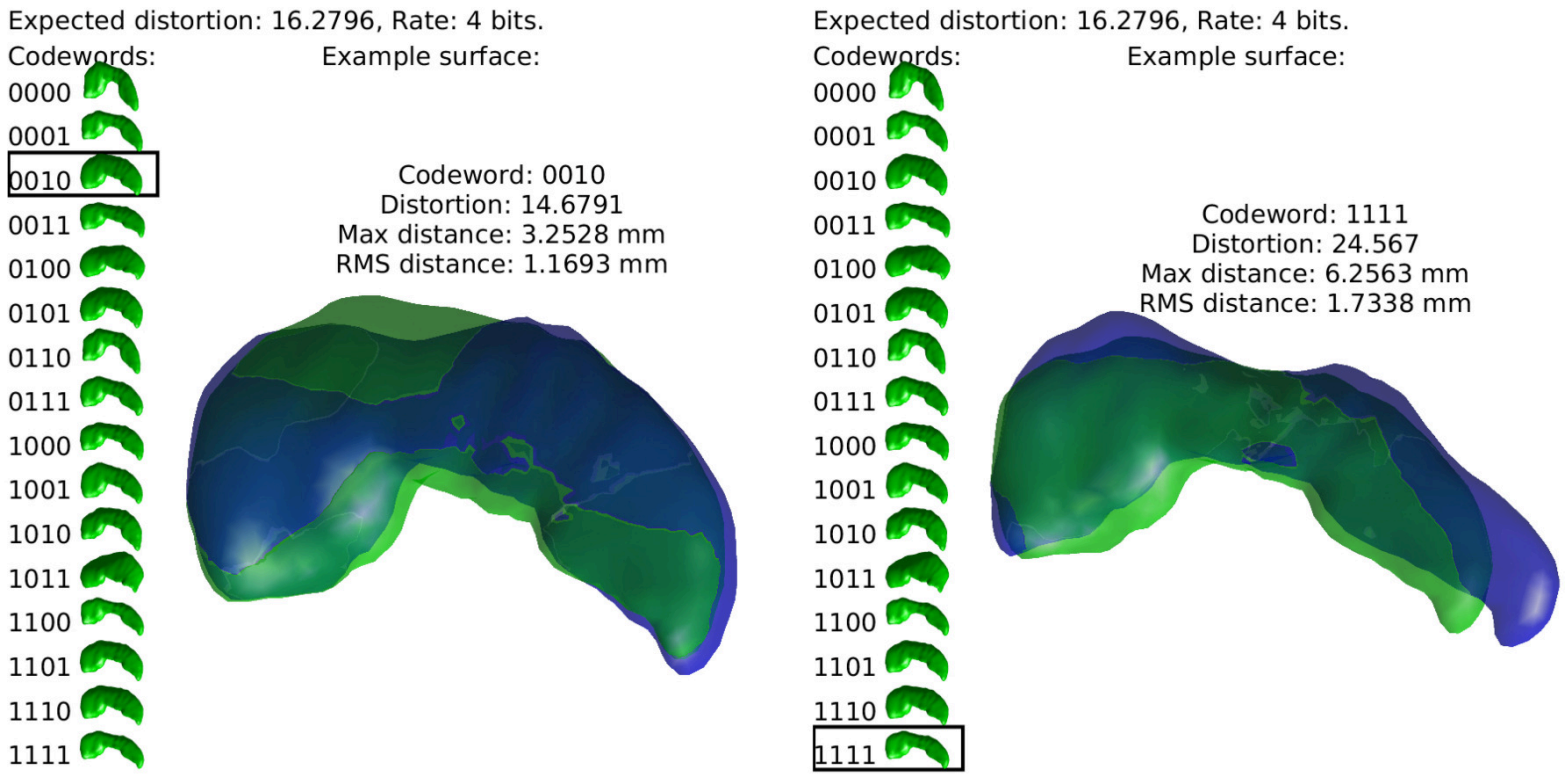
The idea of the discrete coding is illustrated. Codewords, random realizations of anatomy, are shown at left in green. Two examples of real hippocampi are shown in blue, with their closest codewords overlayed in green.

Our approach is to build sets of “codewords,” specific examples of anatomical structures, and to encode a newly observed anatomy as one these words. This continuous to discrete process necessarily introduces distortion, and the relationship between the number of codewords required (the rate of our code) and this distortion measure is studied through rate distortion theory. By relating distortion to geometric error, we can establish the code rate required for errors at a certain spatial scale. This idea is illustrated in Figure 1, using a simple example of describing the hippocampus with a four bit code. In what follows we describe how this procedure is used to characterize the complexity of human anatomy at clinically relevant scales.

Much of the existing work in computational anatomy has focused on addressing the complexity of human anatomy through data reduction techniques. Foremost, the object of study was moved from high dimensional images to smooth diffeomorphisms via the random orbit model, with a fixed template (Miller et al., 1997) or several templates (Tang et al., 2013). Later, the construction of diffeomorphisms, typically created from a time varying velocity field, was moved to an initial velocity, with dynamics fixed via a conservation of momentum law (Miller et al., 2006). Sparsity was introduced, both optimized for specific data types (Miller et al., 2006), and for ease of interpretation and computational burden (Durrleman et al., 2014). Further, low dimensional models were developed based on empirical distributions such as PCA (Vaillant et al., 2004), or linear discriminant analysis (see Tang et al., 2014 for one example), or other techniques such as locally linear embedding (Yang et al., 2011). Instead of continuing the trend of *dimensionality reduction*, the novelty of this work is to address *discretization*. Our specific contribution is to develop a coding procedure informed by Bayesian priors, opening the study of anatomy through medical imaging to information theoretic techniques, and for the first time estimate the entropy of a population of neuroanatomy.

## 2. Methods

### 2.1. Empirical priors

Data used in the preparation of this article were obtained from the Alzheimer's Disease Neuroimaging Initiative (ADNI) database (adni.loni.usc.edu). The ADNI was launched in 2003 as a public-private partnership, led by Principal Investigator Michael W. Weiner, MD. The primary goal of ADNI has been to test whether serial magnetic resonance imaging (MRI), positron emission tomography (PET), other biological markers, and clinical and neuropsychological assessment can be combined to measure the progression of mild cognitive impairment (MCI) and early Alzheimer's disease (AD). For up-to-date information, see www.adni-info.org.

**Figure 2:**
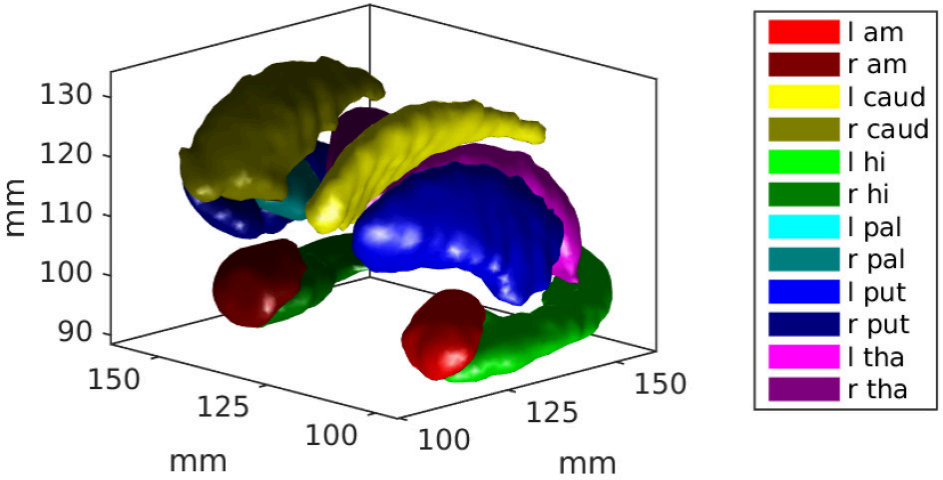
An example of the subcortical gray matter structures studied in this work are shown. They include left and right amygdala, caudate, hippocampus, globus pallidus, putamen, and thalamus.

Using 650 brains from the ADNI and the Open Access Series of Imaging Studies (OASIS), we extract 12 subcortical gray matter structures (left and right amygdala, caudate, hippocampus, globus pallidus, putamen, and thalamus) using FreeSurfer (Fischl et al., 2002) and create triangulated surfaces. For each structure, population surface templates were estimated following (Ma et al., 2010), and diffeomorphic mappings from template to each target were computed using current matching (Vaillant and Glaunès, 2005). The subcortical structure surface templates are shown in Figure 2.

These datasets were combined to provide a larger and more diverse sample. This is useful for achieving our goal of characterizing a population, as opposed to using more well controlled samples for hypothesis testing between populations.

As described in Miller et al. (2006), these diffeomorphic transformations are parameterized by an initial momentum vector, with three components per triangulated surface vertex at point *x*^*i*^ ∈ ℝ^3^ denoted by $p_{0}^{i}$. This momentum defines a smooth velocity field *v* which is integrated over time to construct diffeomorphisms φ, as described by the following system of equations.

(2) $$\begin{array}{r} v\left(x\right)=\underset{i}{\sum }K\left(x-x^{i}\right)p^{i} \end{array}$$

(3) $$\begin{array}{r} {ẋ}^{i}=v\left(x^{i}\right),\;x_{0}=\text{template} \end{array}$$

(4) $$\begin{array}{r} {ṗ}^{i}=-Dv^{T}\left(x^{i}\right)p^{i} \end{array}$$

(5) $$\begin{array}{r} \dot{\varphi }=v\left(\varphi \right),\;\varphi_{0}=\text{identity}, \end{array}$$

where *K* is a Gaussian kernel of standard deviation 6.5 mm. The space of possible parameterizations is a vector space, in the sense that it is closed under scalar multiplication and addition. This substantial difference from the diffeomorphisms themselves, which are only closed under composition, allows us to study shape using multivariate Gaussian models.

The initial momentum vectors are analyzed using tangent space PCA as proposed in Vaillant et al. (2004), and described for this population in Tward et al. (2013). A low, *B* dimensional representation is chosen by selecting the largest principal components that account for 95% of the trace of the covariance matrix. The low dimensional approximation of our initial momentum vector *p*_0_ is written

$$\begin{array}{l} p_{0}=b^{0}+\overset{B}{\underset{i=1}{\sum }}\beta^{i}b^{i} \end{array}$$

where $p_{0},b^{0},b^{i}$ are vectors of dimension three times the number of vertices, and β^*i*^ are scalar parameters. As described in the references, the basis vectors *b*^*i*^ are chosen to be orthonormal with respect to an inner product in the dual space of smooth functions, $\langle b^{i},b^{j}\rangle =\underset{k}{\sum }b^{ikT}K\left(x_{0}^{i},x_{0}^{j}\right)b^{jk}=\delta_{ij}$, where *T* denotes the transpose of a vector in ℝ^3^, and δ_*ij*_ is the Kronecker delta (1 if *i* = *j* and 0 otherwise).

Our empirical prior model corresponds to choosing the β^*i*^ as independent Gaussian random variables with mean 0 and variance σ^2*i*^, measured from the population. We create one empirical prior for each of the 12 subcortical structures examined.

### 2.2. Rate distortion theory for multivariate Gaussians

For readers unfamiliar with rate distortion theory we review some standard terminology and results which will be necessary for our purposes. More details can be found in Cover and Thomas (2012).

Our empirical prior is a continuous distribution and must be discretized to be understood in terms entropy and complexity. This can be achieved through *encoding* our continuous random vectors β^*i*^. That is, through constructing a mapping *e*(β) from β ∈ ℝ^*B*^ to a finite set *S*. Here *S* is chosen to be the set of binary strings of fixed length, as shown in the left side of each subfigure in Figure 1. Associated to this encoder is a *decoder*, a mapping *e*(*s*) from *s* ∈ *S* back to ℝ^*B*^. Because *S* is finite, *d*(*e*(β)) can take only a finite number of values in ℝ^*b*^, which we enumerate as ${\hat{\beta }}^{i}$ for positive integers *i* and refer to as *codewords*. The distribution of *d*(*e*(β)) is therefore a weighted sum of Dirac measures at these specific codewords ${\hat{\beta }}^{i}$. Examples of anatomies represented by a set of 16 codewords are shown toward the left side of each subfigure in Figure 1.

One can reason that an encoder/decoder pair is good if β is similar to *d*(*e*(β)) on average. The difference between the two is known as *distortion*. Because it admits well characterized solutions, we measure distortion using sum of square error in this work. Distortion can be minimized if we discretize β by mapping it to its closest codeword. In other words, we choose the encoder by

$$\begin{array}{l} e\left(\beta \right)=s_{i},\text{the}i^{\text{th}}\text{string in}S,\\ \text{where }i=\arg\underset{j}{\min}|\beta -{\hat{\beta }}^{j}{|}^{2}, \end{array}$$

for |·|^2^ the norm squared in ℝ^*B*^, and the decoder by

$$\begin{array}{l} d\left(s_{i}\right)={\hat{\beta }}^{i}. \end{array}$$

Furthermore, one notices that lower distortion can be achieved with larger sets *S*. We refer to the size of *S* as |*S*| = 2^*R*^ for a code *rate R*. We note that *R* is the length of the binary strings in *S*, so that the examples in Figure 1 have a rate of *R* = 4 bits.

We aim to identify the minimum number of codewords that are required to achieve a given amount of expected distortion *D*. The best achievable code is characterized by the *rate distortion curve* (*D* as a function of *R*). This can be shown to be equal to the minimum of the mutual information between β and *d*(*e*(β)) while enforcing distortion less than or equal to *D* (i.e., the shortest code respecting the distortion constraints is the worst one: that with the smallest mutual information with β). This definition, while arcane, can be used to compute rate distortion curves in closed form in several situations. In general this curve can be approached asymptotically, by coding blocks of *N* structures simultaneously using 2^*NR*^ codewords, considering the average distortion, and letting *N* → ∞.

The details of Gaussian rate distortion curves can be found in Cover and Thomas (2012) chapter 13. For single variate Gaussian random variables with square error distortion the rate distortion curve can be computed in closed form:

$$\begin{array}{l} R(D)=\{\begin{array}{ll} \frac{1}{2}\log_{2}\frac{\sigma^{2}}{D}, & 0\leq D\leq \sigma^{2}\\ 0, & D>\sigma^{2} \end{array} \end{array}$$

Note that if the desired distortion is greater than the variance, we need only 1 codeword, or *R* = 0. If this 1 codeword is the mean, the expected distortion is equal to the variance. Otherwise, we require more codewords in a manner increasing logarithmically with the variance.

We finally specify how our codewords are chosen. This minimal distortion can be achieved for codewords chosen as independent realizations of a Gaussian random variable. We can motivate this as follows. Let the joint distribution of data β and codewords $\hat{\beta }$ be described by drawing β from the distribution $\hat{\beta }\sim N\left(0,\sigma^{2}-D\right)$, and $\beta =\hat{\beta }+err$ with error err~N(0,D). This coding scheme has square error distortion at most *D*. The mutual information between β and $\hat{\beta }$ can be calculated as $\frac{1}{2}\log\frac{\sigma^{2}}{D}$, the value of the rate distortion curve. On the other hand, if the allowable distortion *D* > σ^2^, we can simply choose $\hat{\beta }=0$ and achieve *R*(*D*) = 0.

This approach can be extended to *B* independent Gaussians using the *reverse water filling* method.

$$\begin{array}{l} D_{i}=\{\begin{array}{ll} \lambda , & \lambda <\sigma_{i}^{2}\\ \sigma_{i}^{2}, & \lambda \geq \sigma_{i}^{2} \end{array}\\ \lambda \text{ s}.\text{t}.\;\sum_{i=1}^{B}D_{i}=D \end{array}$$

The optimum corresponds to choosing a fixed amount of distortion per dimension for variables with “large” variance ($\sigma_{i}^{2}>\lambda$), and no additional codewords for those of “small” variance.

This leads to the rate distortion curve

(6) $$\begin{array}{r} R\left(D\right)=\overset{B}{\underset{i=1}{\sum }}\frac{1}{2}\log\frac{\sigma_{i}^{2}}{D_{i}} \end{array}$$

which can be asymptotically approached (coding blocks of *N* anatomies simultaneously, and allowing *N* → ∞) with a random code, with the *i*th component of a codeword generated according to

$$\begin{array}{l} {\hat{\beta }}^{i}\sim \{\begin{array}{ll} N(0,\sigma_{i}^{2}-\lambda ), & \sigma_{i}^{2}\geq \lambda \\ N(0,0), & \sigma_{i}^{2}<\lambda \end{array} \end{array}$$

The reverse waterfilling method is named by imagining each independent Gaussian to be represented by an object of height $\sigma_{i}^{2}$ in a room with rising water. As the water rises, those Gaussians with small variance become submerged. Everything below the surface represents distortion, a fixed amount for each of the variables with large variance, and amount equal to its variance for the others. We allow the water to continue to rise until the the total distortion is given by *D*.

For our experiments, from the empirical prior for each subcortical structure a set of codewords is generated for rates from 0 to 32 bits, and for coding *N* = 1 and *N* = 2 examples simultaneously.

### 2.3. Complexity at clinically relevant spatial scales

By shooting our template with the initial momentum from a given codeword, we can compute the expected geometric error between an anatomical structure defined by our continuous model and its discretely coded version. Error in units of mm are considered, using Hausdorff distance between surfaces (max error between closest pairs of vertices between realization and codeword). We measure geometric error as a function of rate, fit this curve to a simple model, and compute the code rate required at clinically relevant scales. Owing to the computational complexity of looping through 2^32^ codewords and solving system Equation (2), this procedure is repeated for 10 observations of each subcortical structure.

## 3. Results

### 3.1. Empirical priors

**Figure 3:**
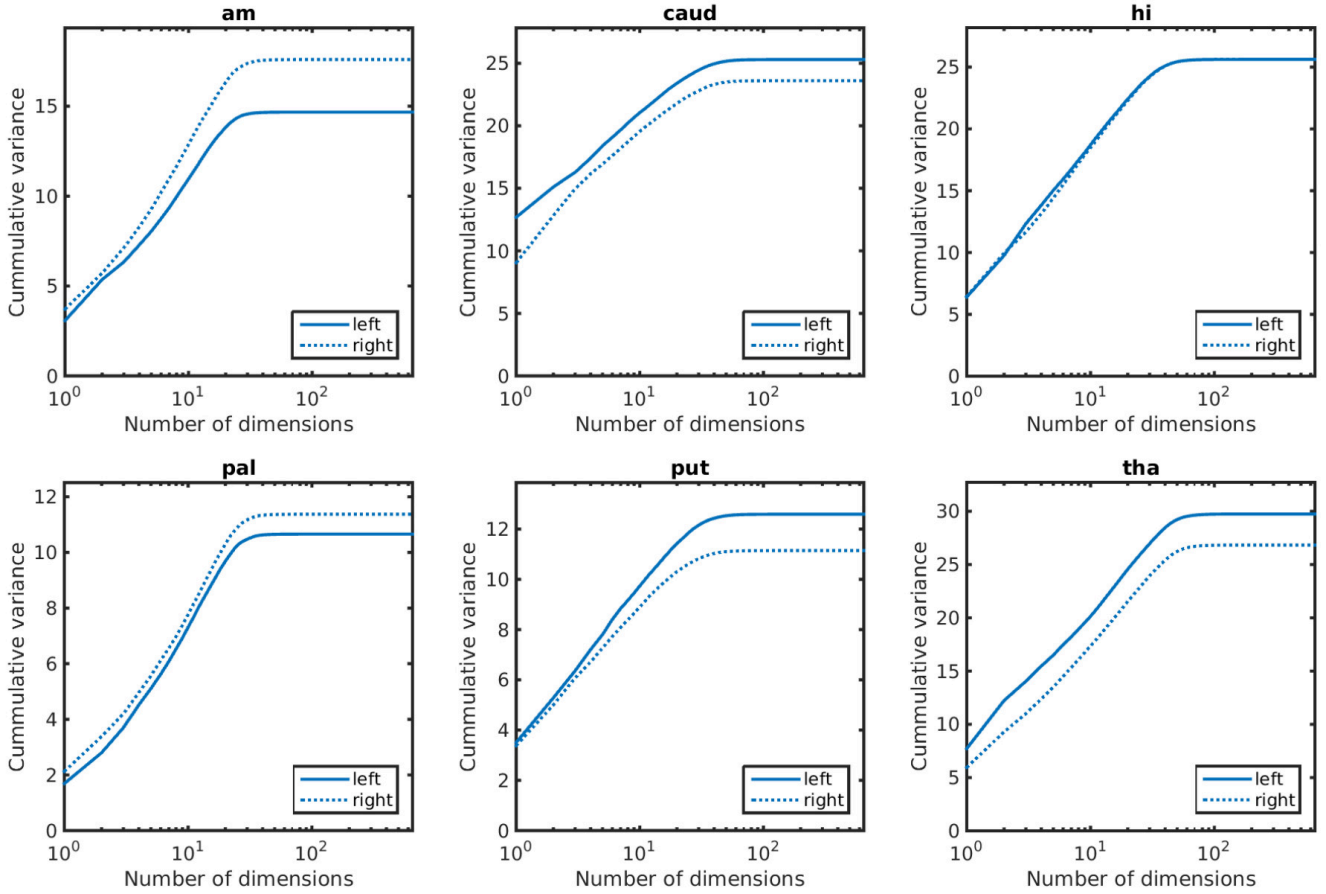
Cummulative variance as a function of dimensions for anatomical priors. In lexicographic order: amygdala, caudate, hippocampus, globus pallidus, putamen, thalamus.

**Figure 4:**
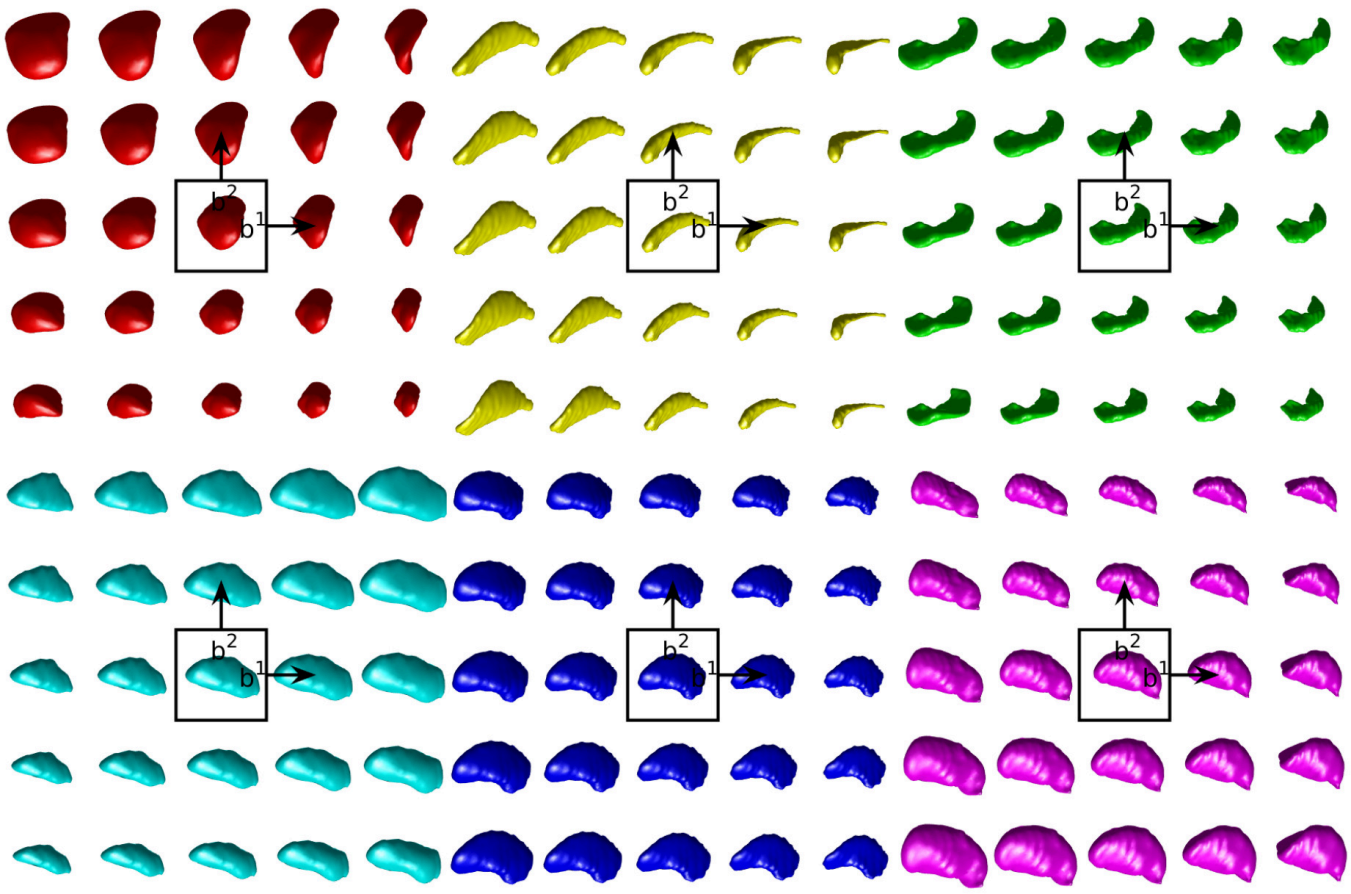
Examples of the first two modes of variability in our empirical prior for left side structures. The mean shape is shown in the center. Each step to the right (top) moves one standard deviation in the direction of the first (second) mode of variation. In lexicographic order: amygdala, caudate, hippocampus, globus pallidus, putamen, thalamus.

Empirical prior models for the 6 structures examined are quantified in terms of their variance spectra in Figure 3. The number of dimensions that captured 95% of the trace of the covariance matrix for each left (right) structure was found to be: amygdala 21 (22), caudate 26 (26), hippocampus 31 (32), globus pallidus 24 (24), putamen 27 (25), thalamus 39 (41). These numbers are quite similar for the left and right hand sides of the same structure. Examples of the first two modes of variability are shown for the left side structures in Figure 4.

### 3.2. Rate distortion calculations

**Figure 5:**
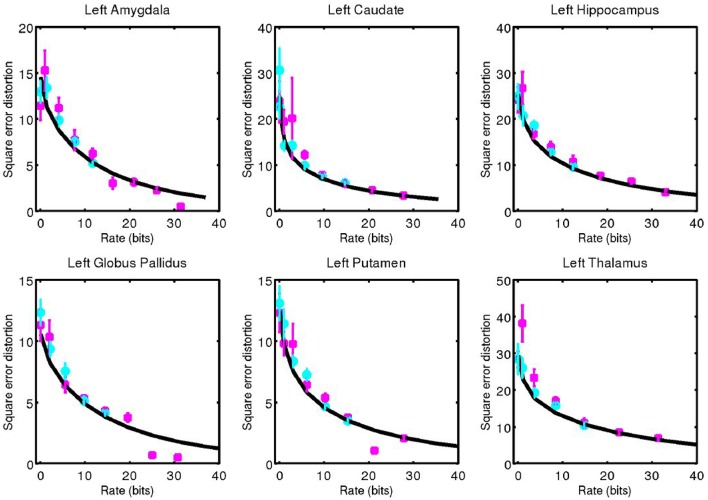
Square error distortion as a function of code rate for left side structures. Coding one structure is shown in magenta, and two structures simultaneously is shown in cyan. The rate distortion curve for a multivariate Gaussian model is shown in black.

**Figure 6:**
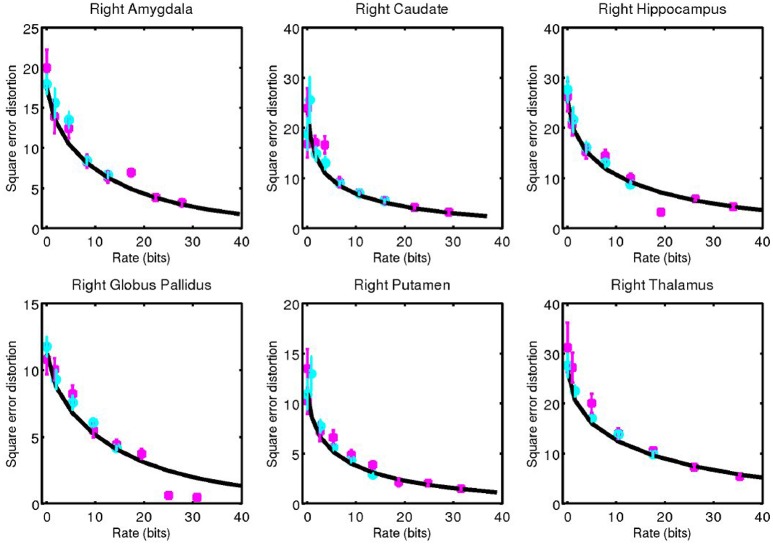
Corresponding data from Figure 5 for right side structures.

For each subcortical structure we calculate square error distortion as a function of code rate. For coding one structure at a time, we use codes with rate from 0 to 32 bits. For coding two structures at a time, we use codes with rate from 0 to 16 bits. The results of these calculations are shown for left side structures in Figure 5 and for right side structures in Figure 6. Mean and standard error for coding one structure is shown in magenta, and that for two structures simultaneously is shown in cyan. The two results are seen to be similar, indicating that not much is gained by encoding several structures simultaneously, since the coefficients β are already high (as compared to 1) dimensional. For each structure, we calculate the rate distortion curve described by Equation (6) from the corresponding multivariate Gaussian. This represents a lower bound on the expected value of the data shown. That our data is close to these curves serves as an indication that our procedure is valid.

### 3.3. Complexity at clinical scale

**Figure 7:**
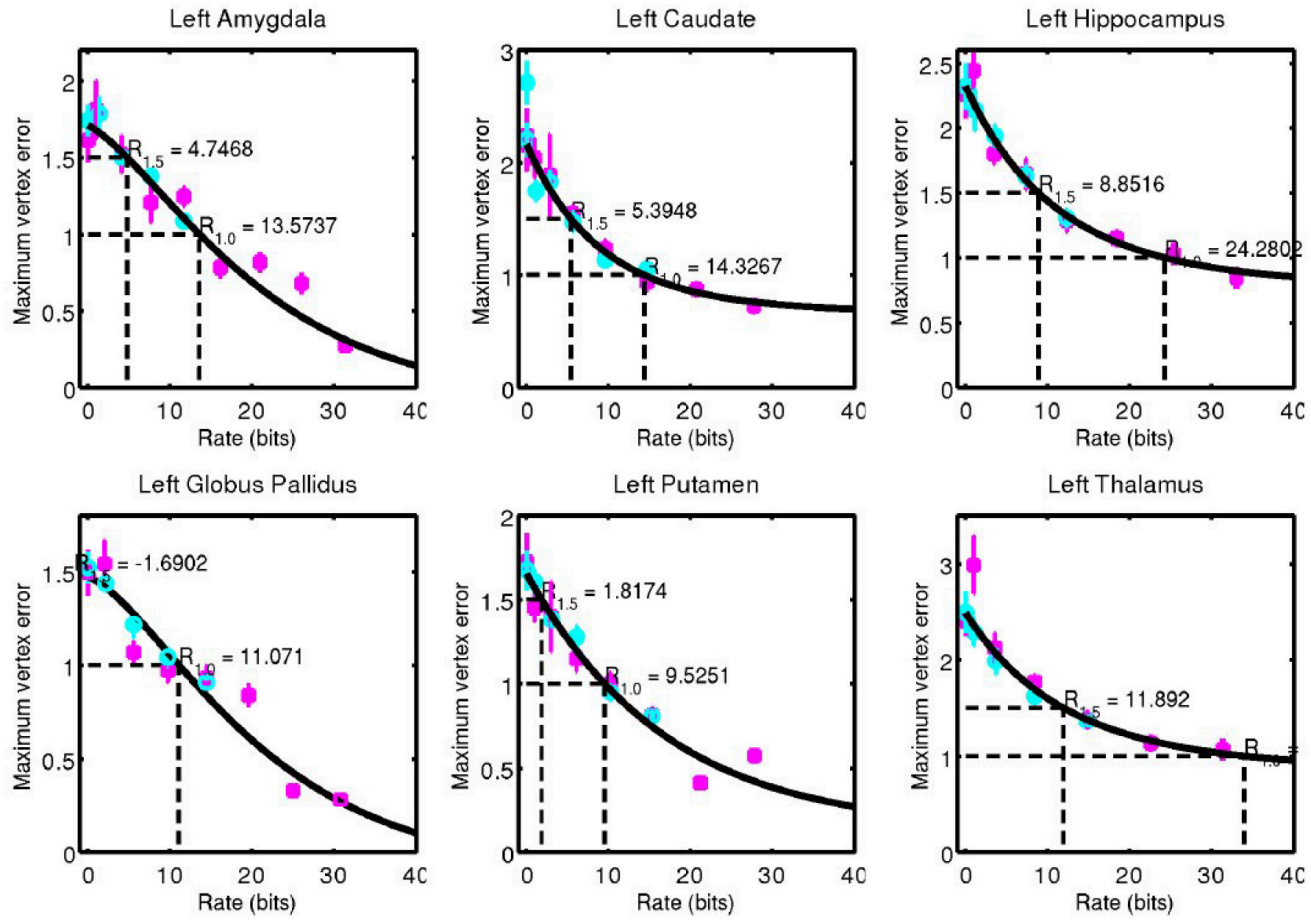
Hausdorff distance between example surfaces and closest codeword. Coding one structure is shown in magenta, and two structures simultaneously is shown in cyan. The black curve is a simple fit through the data (not a model), and is used for estimating code rate at 1 and 1.5 mm geometric error.

**Figure 8:**
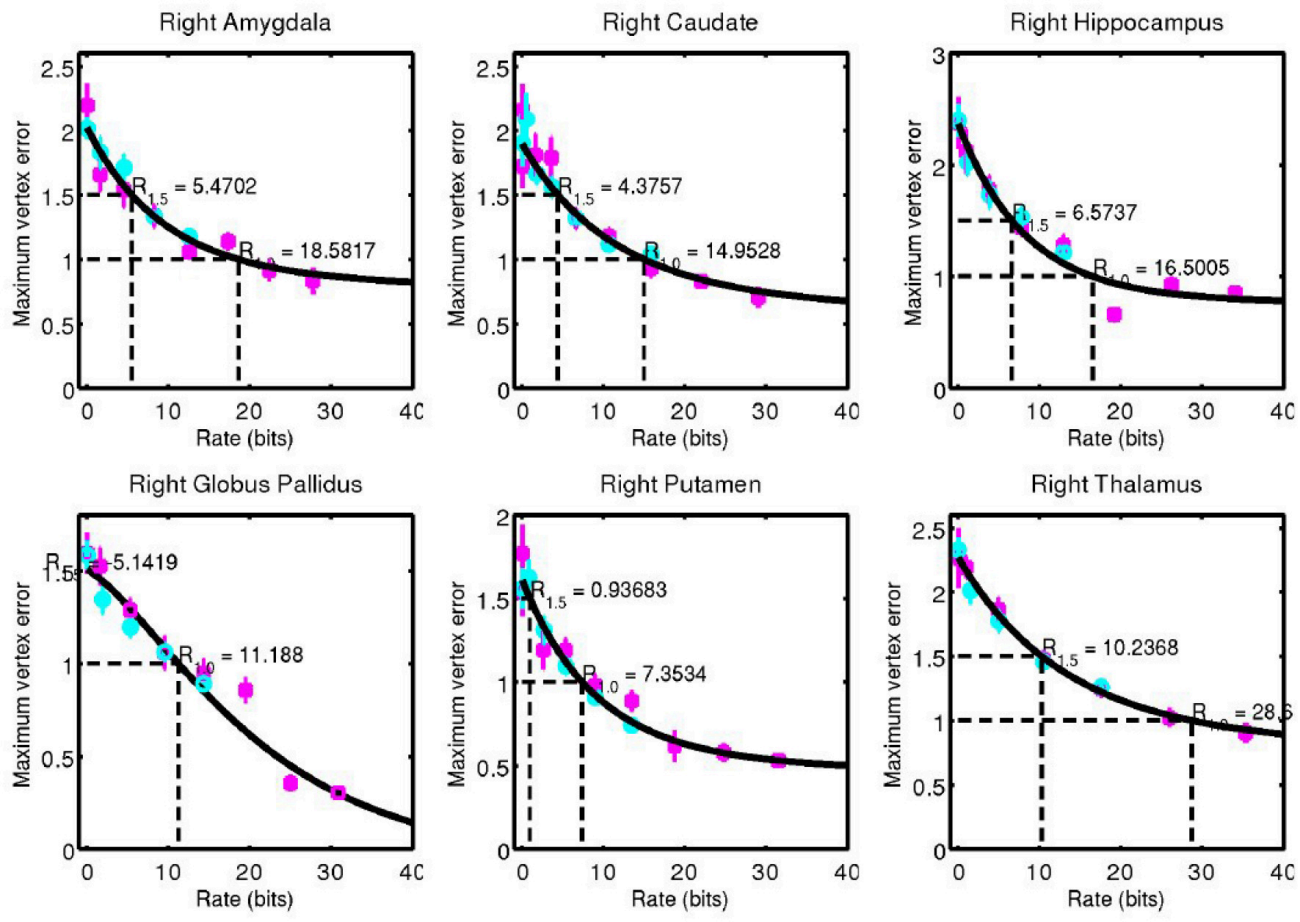
Corresponding data from Figure 7 for right side structures.

For each structure examined, we consider the geometric error between our codeword and the anatomy they represent. We quantified this through the Hausdorff distance between triangulated surfaces. Mean and standard error of this data is shown for left side structures in Figure 7 and for right side structures in Figure 8.

**Figure 9:**
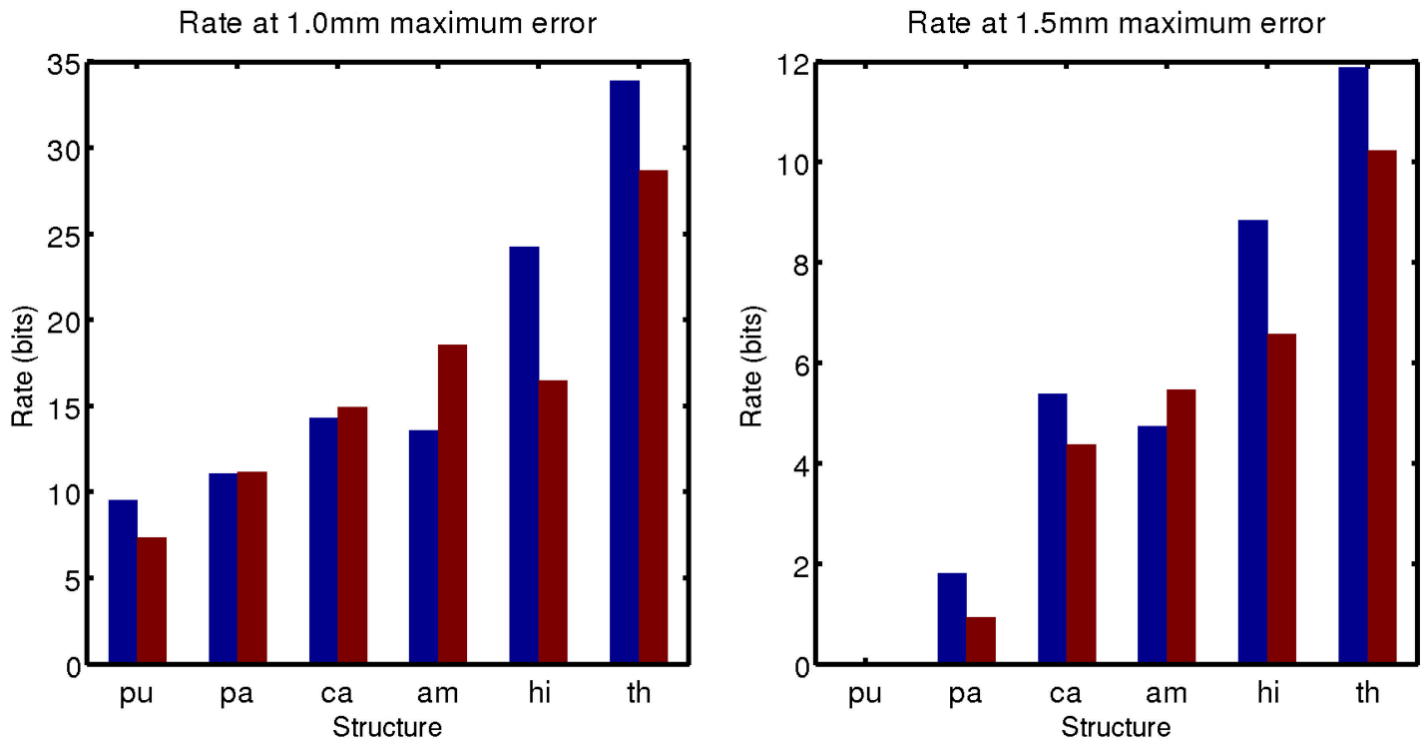
Code rate required for 1 mm **(Left)** and 1.5 mm **(Right)** geometric error.

A simple curve was fit through the data and used to estimate the code rate required for 1 and 1.5 mm of maximum error, values that are on the order of 1 voxel in a typical clinical MRI. These rates are shown in Figure 9.

## 4. Conclusion

The complexity of the subcortical gray matter structures we have examined range from the order of 5–35 bits for 1.0 mm geometric error, and 0–12 bits for 1.5 mm geometric error. Note that at 1.5 mm error, a 0 bit code is sufficient for the putamen. Its low amount of variability means it can be represented by an average template only at this accuracy.

While using up to 2^32^, or more than 4 billion, codewords may seem excessive, this still represents a huge amount of data compression. Binary segmentation images, contain roughly 100^3^ voxels, or the order of one million bits. The triangulated surfaces have roughly 1,000 vertices, each component stored to double precision, which correspond to about 192,000 bits. We have shown that 32 bits, or an amount of data equivalent to one single precision floating point number, is enough to encode the variability of gray matter subcortical structures at clinically relevant spatial scales.

The potential for this work to impact clinical practice stems from the fact that entropy can be used to devise lower bounds on the variance of estimators, and that information can be used as an important figure of merit. When this work is extended to considering mutual information between anatomical form and diagnostic status, it could directly influence clinical decision making and optimization of imaging procedures.

For example, the Image Gently campaign (Goske et al., 2008), a program designed to reduce radiation exposure to pediatric patients, suggests first to “reduce or ‘child-size’ the amount of radiation used” and second to “scan only when necessary” through a discussion of a risk-benefit ratio. Because lower radiation doses can be used at lower resolution, the analysis presented as a function of resolution could lead to appropriately choosing a dose level for a given level of certainty required. Further, a scan could be avoided if it will not reduce entropy about diagnostic status sufficiently.

Turning to imaging optimization, task based analysis of image quality (Sharp et al., 1996) has been used for many years, but figures of merit have been largely designed to reflect the performance of idealized observers on simple detection or estimation tasks (Barrett et al., 1995). Anatomical variability is often described simply as stationary power law noise (see for example Burgess, 1999). Mutual information between observed anatomy and diagnostic status could be used as a figure of merit for system design that appropriately accounts for anatomical variation and models realistic imaging tasks.

One limitation of this study is that we have encoded only a small number of structures. Due to the computational complexity of searching through each codeword and solving a high dimensional geodesic shooting equation in each case, we limited the number examined. As this work progresses, we will include larger samples. In what follows, we will restrict ourselves to disease specific populations to measure how entropy changes with disease state. This will enable calculation of the mutual information between anatomical phenotype and disease state as shown in Equation (1).

## Author contributions

DT and MM developed the approach and planned experiments. DT developed tools for computational analysis.

### Conflict of interest statement

MM reports personal fees from AnatomyWorks, LLC, outside the submitted work and jointly owns AnatomyWorks. This arrangement is being managed by the Johns Hopkins University in accordance with its conflict of interest policies. MM's relationship with AnatomyWorks is being handled under full disclosure by the Johns Hopkins University. The other authors declare that the research was conducted in the absence of any commercial or financial relationships that could be construed as a potential conflict of interest.
